# Polyvinylidene Fluoride Surface Polarization Enhancement for Liquid-Solid Triboelectric Nanogenerator and Its Application

**DOI:** 10.3390/polym14050960

**Published:** 2022-02-28

**Authors:** Duy Linh Vu, Chau Duy Le, Kyoung Kwan Ahn

**Affiliations:** Fluid Power & Machine Intelligence (FPMI) Laboratory, School of Mechanical Engineering, University of Ulsan, 93, Daehak-ro, Nam-gu, Ulsan 44610, Korea; vuduylinhbk@gmail.com (D.L.V.); lechauduy@gmail.com (C.D.L.)

**Keywords:** polyvinylidene fluoride, surface polarization, triboelectric nanogenerator, self-powered, streamflow

## Abstract

Liquid-solid triboelectric nanogenerator (TENG) has been great attention as a promising electricity generation method for renewable energy sources and self-powered electronic devices. Thus, enhancing TENG performance is a critical issue to be concerned for both practical and industrial applications. Hence in this study, a high-output liquid-solid TENG is proposed using a polyvinylidene fluoride surface polarization enhancement (PSPE) for self-powered streamflow sensing, which shows many advantages, such as adapt to the sensor energy requirement, multiple parameters sensing at the same time, eliminate the influence of ion concentration. The TENG based on PSPE film has the maximum power density of 15.6 mW/m^2^, which is increased by about 4.7 times compared to commercial PVDF-based TENG. This could be attributed to the increase of the dielectric constant and hydrophobic property of the PVDF film after the surface polarization enhancement process. Furthermore, the PSPE-TENG-driven sensor can simultaneously monitor both the physical and chemical parameters of the streamflow with high sensitivity and minimum error detection, which proves that the PSPE-TENG has enormous potential applications in self-powered streamflow sensing.

## 1. Introduction

Recently, triboelectric nanogenerator (TENG) has been great attention as a promising electricity generation method for renewable energy sources, owing to their advantages such as easily fabricated device, cost-effectiveness, low weight, portability, and miniaturization [1,2,3,4]. Especially, TENGs offer an availability-to-applications as sensors and actuators by using the directly affecting of the output parameters onto the electrical signal [5,6,7]. Particularly, liquid-solid TENGs, which can harvests energy from diverse water resources, for example, water streams [8], raindrops [9,10], tidal waves [11], have been developed as self-powered sensors in streamflow, human motion, chemical property, and force/pressure [12,13,14,15,16].

Streamflow, which is widely used to describe a flow type of streams, rivers, and other channels, is the most important component of the water cycle [17,18]. Therefore, the study of streamflow plays a vital role in water energy harvesting, mass/heat transfer, environmental pollution, and early warning of natural disasters [19,20]. Consequently, various streamflow sensing devices have been developed to meet the demand for accurate water flow spectrum detection based on an ultrasonic wave, electromotive force, pressure drop, capacitance changing. However, streamflow properties research is far more challenging due to the complicated of the flow leading to the specific dedication for the existing sensors system, such as complex structure, signal distortion, real-time response, flaws in millimeter-scale pipelines [21,22,23]. Moreover, most sensor devices need batteries as energy supply, which partially restricts their application due to non-negligible replacement costs, environmental concerns, and limited lifetime.

Instead of conventional streamflow spectrum sensors, a streamflow sensor based on TENG might be deemed the next-generation streamflow sensor due to its self-power, high portability, and sensitivity [24]. The ability to generate enough output power of TENGs, which is normally stored in a capacitor or a battery, is critical to their success in practical applications. However, due to the mediocre quality of materials that make up liquid-solid TENGs, their output performance is insufficient to power electronic devices [25]. Thus, it is necessary to improve the output performance of the liquid-solid TENG based on raising the transferred charge density from liquid-solid electrification introduction.

There are numerous strategies to enhance the transferred charge density of the liquid-solid TENG but most of them have depended on the changing of triboelectric layer properties, such as large dielectric constant [26,27,28], tunable tribopolarity [29], high hydrophobicity [30,31], and injecting electrical charges [32]. For example, Shao et al. used BaTiO_3_ to improve the dielectric constant of the cellulose film, so that the transfer charge of 13.5% vol BaTiO_3_/cellulose reached 76.5 nC, which increases about 2 times compared to pristine cellulose [27]. Jang et al. tried to increase the output performance of TENG by injecting electrical charges on the triboelectric surface through monocharged electret [32]. It was possible to produce a peak power gain of over 1000 times after intentionally injecting electrical charges. Moreover, many studies have effectively proved the increase in transfer charge by fabricating superhydrophobic films. It is possible to conclude that the output performance of TENG is increased through the above strategies, however, they require complex additional conditions, such as high electric field, high-temperature process, high technology equipment [33,34]

In this study, we propose a simple approach for fabricating polyvinylidene fluoride (PVDF) film with an outstanding dielectric constant and hydrophobic property through a surface polarization enhancement process that can be used as a triboelectric layer to put forward a high-output liquid-solid TENG. The silica nanoparticles (SiNPs) and 1H,1H,2H,2H-Perfluorooctyltriethoxysilane (POTS) are in turn grown on the surface of the PVDF film by using the dip-coated process to form the PVDF surface polarization enhancement (PSPE) film. When compared to a TENG made from a commercial PVDF film, the output performance of PSPE-TENG was substantially greater, with the highest value of 15.6 mW/m^2^, which is approximately 4.7 times higher. Considering the capability of immediately transforming the mechanical energy of streamflow into the electric signal of the PSPE-TENG, this TENG device could be constructed as a self-powered streamflow sensor. The output current and voltage of the PSPE-TENG sensor demonstrate a strong linear relationship and a high sensitivity with the streamflow properties, such as flow rate, ion concentration. Furthermore, based on its simple design and excellent performance, this sensor would have enough power for its self-activity, which would be critical in the future development of self-powered streamflow sensors.

## 2. Materials and Methods

### 2.1. Materials

A flat sheet PVDF film (average thickness of 50 μm, pore size of 45 μm), silica nanoparticles (SiNPs, 10–20 nm), and 1,3,5-Benzenetricarbonyl trichloride (TMC, 98%) were purchased from Sigma-Aldrich, St. Louis, MO, USA. 1H,1H,2H,2H-Perfluorooctyltriethoxysilane (POTS, 98%) was acquired from Gelest, Morrisville, PA, USA. Sodium hydroxide, hexane was acquired from Daejung, Siheung-si, Korea. Copper tape, copper conductor, and Kapton tape were purchased from the local market.

### 2.2. Surface Treatment of PVDF Film

**Figure 1:**
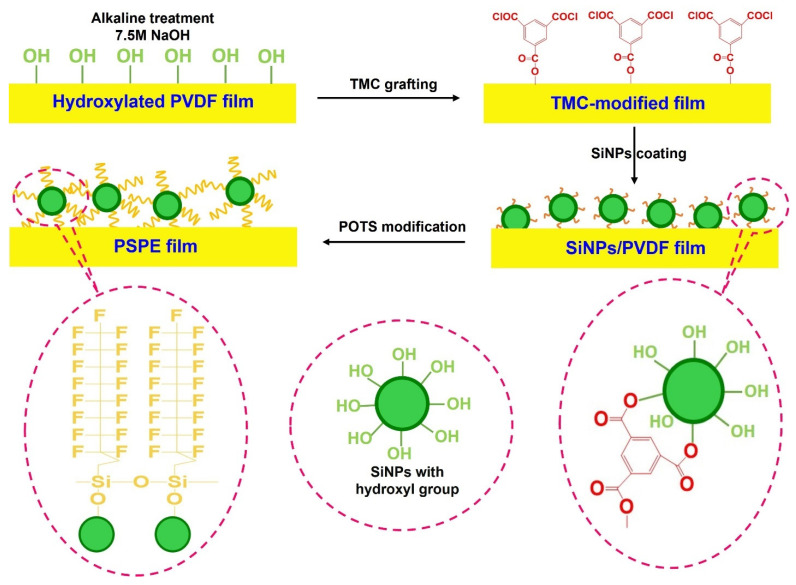
The procedure diagram of the PVDF surface polarization enhancement through SiNPs coated and POTS functionalized.

Figure 1 describes the surface polarization enhancement of PVDF film through SiNPs coated and POTS functionalized. Before the coating process, PVDF film was generated hydroxyl surface functionality by alkaline (7.5 M NaOH) treatment. Thereafter, the hydroxylate film was grafted TMC on the film surface. The TMC-modified film was then soaked in an 0.1 wt% SiNPs solution at 25 °C for 1 h, where SiNPs and TMC make the chemical bonding to form the SiNPs/PVDF film. Subsequently, SiNPs/PVDF film was fluorinated by being immersed in 1.0 wt% POTS solution. After continuous treatment for about 24 h and drying in an oven at 60 °C for 6 h, the PVDF surface polarization enhancement (PSPE) was obtained.

### 2.3. Fabrication of PSPE TENG Device

Single electrode mode TENG devices were assembled using PVDF and PSPE films as triboelectric layers. The PVDF and PSPE films were cut into 20 mm × 20 mm and attached to the copper tapes that were employed as the electrode layer. Then the TENG devices were transferred to the glass sheet, which was used as the supporting substrates.

### 2.4. Characterization and Measurements

The surface morphology of each film was investigated by field emission scanning electron microscopy (FESEM, JSM-6500F, JEOL, Tokyo, Japan). The surface roughness of the film was analyzed by atomic force microscope (AFM, MFP-3D Stand Alone, Oxford Instruments, Abingdon, UK). The Fourier transform infrared spectroscopy (FTIR, Nicolet iS5 FTIR Spectrometer, Thermo Fisher Scientific, Boston, MA, USA) and X-ray photoelectron spectroscopy (XPS, ESCALB-MKII250, Thermo Fisher Scientific, Boston, MA, USA) was used to analyze the chemical structure of the film. The dielectric constant and dielectric loss of the film was measured by an impedance analyzer (3522-50 LCR Meter, Hioki, Nagano, Japan) over the frequency range of 10^3^ to 10^7^ Hz. The hydrophobicity of the film was investigated through the water contact angle by the sessile drop method (SmartDrop, FemtoFAB, Waltham, MA, USA). The output properties of the TENG devices were measured using a Keithley Digital Multimeter (DMM7510, Keithley, OH, USA).

## 3. Results and Discussions

### 3.1. Characterization of PSPE Film

**Figure 2:**
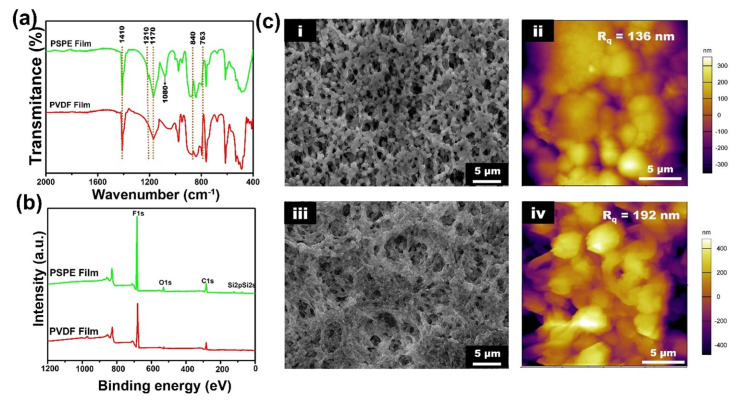
(**a**) FTIR spectra and (**b**) XPS spectra of PVDF and PSPE film. FE-SEM and AFM image of (**c**-**i**,**ii**) PVDF film surface and (**c**-**iii**,**iv**) PSPE film surface.

In order to confirm the polarization enhancement of the PVDF film before and after treatment, FTIR spectra and XPS spectra were used to inspect the chemical structure of PVDF and PSPE film as shown in Figure 2a,b. The FTIR spectra of PVDF film exhibited broad bands at 1410, 1210, and 1170 cm^−1^ are referred to CH_2_ vibration, symmetrical CF_2_ stretching, and asymmetrical CF_2_ stretching, separately. In addition, the band located at 840 and 763 cm^−1^ indicated the crystalline PVDF β-phase and α-phase [35]. Otherwise, the silicon group was found at the newly appeared band of 1080 cm^−1^, suggesting the success of chemical bonding formation between TMC and SiNPs on the film surface [36]. Moreover, it is clearly in Figure 2b that the XPS spectra of the PVDF film only contain the C1s and F1s peaks while O1s and Si2p peaks appear in the scan spectra of the PSPE film. Table 1 summarized that the Si element and O element percentage increased significantly from 0% to 1.58% and 0.45% to 3.64%, indicating a large amount of SiNPs on the film surface. SiNPs not only provide abundant OH groups for the surface fluorinated process but also tailor the surface structure of these films [37]. Therefore, the POTS functionalized has a remarkable effect on the film surface that the F element percentage increases from 48.85% to 53.39% as illustrated in Figure 2b and Table 1. The accomplishment of SiNPs coated and POTS functionalized confirmed the self-assembled organosilane compound layer on the film surface, leading to the increase of the surface polarization.

Figure 2c presents the surface morphology and roughness of the PVDF and PSPE film through FE-SEM and AFM images. It is observed that the FE-SEM image of PVDF film (Figure 2c(i)) exhibits a high uniform and porosity surface. The pore is evenly distributed on the film surface with the sizes appeared around 400 nm to 600 nm. On the contrary, the porosity surface of PSPE film (Figure 2c(iii)) tends to decrease, which can be explained by the hydroxyl surface functionality process. Besides, various nanoscale protrusions represent the SiNPs aggregation on the PSPE film surface were observed in the FE-SEM image due to the strong chemical bonding interaction between these nanoparticles and TMC. The aggregated SiNPs on the film surface was resulting to increase in hydrophobicity owing to the increasing of surface roughness and multiple kinetic barriers supplied [38]. Additionally, the increase of root means square roughness (Rq) of the film was demonstrated as shown in the AFM images (Figure 2c(ii,iv)). It could be observed that after polarization enhancement of the film, the Rq value of the PSPE film increases significantly from 136 nm to 192 nm, which greatly improved the water contact angle of the film.

**Figure 3:**
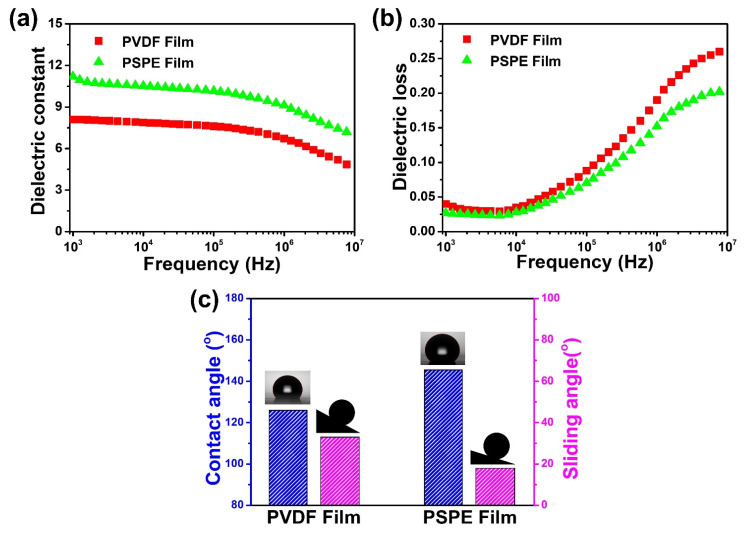
Frequency dependence of (**a**) dielectric constant and (**b**) dielectric loss of PVDF and PSPE films at room temperature; (**c**) contact angle and sliding angle of these films.

In this study, the surface polarization enhancement of PSPE film is mainly due to the increase of the fluorine functional group, which is related to the dielectric constant and surface energy of the solid [37,39,40]. Figure 3a,b presents the dielectric constant and dielectric loss of PVDF and PSPE films as frequency-dependent at room temperature. It seems that the dielectric constant of these films remains constant over frequency from 10^3^ to 10^5^ Hz and then sharply decreases at a higher frequency which is a usual dielectric behavior as other reports [26,41,42]. The dielectric constant of the PSPE film is remarkably higher than that of the PVDF film, especially at low frequency. Notably, at the frequency of 10^3^, the dielectric constant of PSPE film was found approximately 11.2, which is about 40% higher than PVDF. This is due to the fact that the increase of fluorine element, one of the most electronegative elements, is able to increase interfacial polarization. Another reason for the dielectric constant enhancement is the crosslinking of SiNPs on the PVDF surface. The improvement of the dielectric constant exhibits strong capacitance of the films, which means the triboelectric activities can achieve a higher effect. Additionally, the frequency dependence of dielectric loss was found at a low level (~0.03), excluding those in the higher frequency range due to the molecular motion behavior of crystalline PVDF polymer. Correspondingly, low dielectric loss is an excellent property for high-energy-density capacitors, which is a good candidate for triboelectric layer [43].

To investigate the surface energy of the PVDF and PSPE films, the water contact angle and sliding angles were measured and summarized in Figure 3c. The results suggest that the contact angle of the film after treatment increased sharply from 126.3° to 145.5°, while the sliding angles decrease from 33° to 17°. Following Young’s equation [37], the surface energy of the solid is given by:

(1) $$\gamma_{S}=\gamma_{SL}+\gamma_{L}cos\theta_{0\;}$$

where $\theta_{0\;}$ is the contact angle of the smooth surface, $\gamma_{S}$ is the surface energy of the solid, $\gamma_{SL}$ is the interfacial tension of solid-liquid, and $\gamma_{L}$ is the surface tension of the liquid. When the contact angle is higher than 90°, the surface energy of the solid decreases with an increase of contact angle. Moreover, the relation between contact angle and surface roughness can be determined by the Wenzel equation [44]:

(2) $$cos\theta_{\;w}=R_{f}cos\theta_{0\;}$$

where $\theta_{w}$ is the contact angle of the rough solid surface, $R_{f}$ is the ratio of the surface area to its smooth projected area. Based on the Wenzel model, it could be concluded that the water contact angle increases with an increase of surface roughness, which is fitted with the result of surface roughness as presented in the above section.

### 3.2. Working Principle of TENG

**Figure 4:**
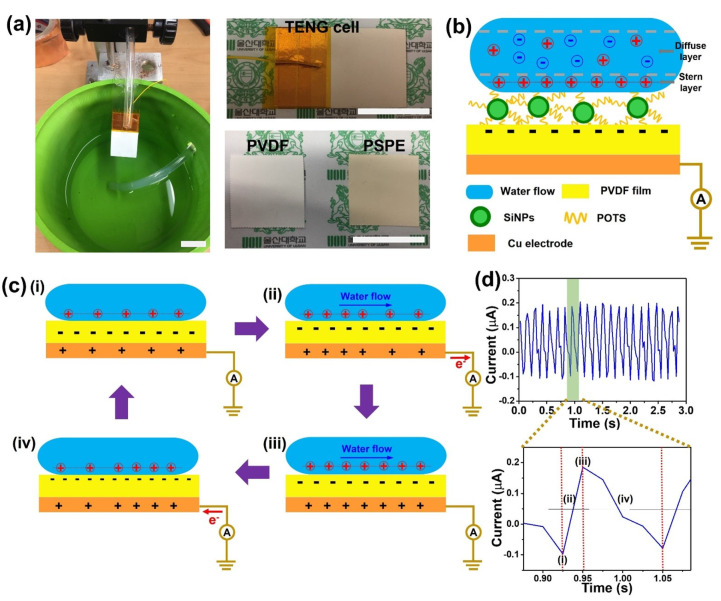
(**a**) The photograph of the TENG system on the left side; the TENG device, PVDF and PSFE film on the right side (scale bars of 2 cm); (**b**) schematic diagram of the TENG structure in contact with water flow; (**c**-**i**–**iv**) the working principle of the TENG; (**d**) Output current of the TENG and magnified view of a cycle showing four states in working principle.

The TENG system is set up following the photograph in Figure 4a. The silicone tube with a diameter of 6.35 mm is used to transport the streamflow on the triboelectric surface of the TENG. Other research also investigated the effect of the inclination angle on the TENG output, the result showed that the inclination angle at 45° gave good stability and high output performance [32]. Therefore, we fixed the inclination angle of the TENG device at 45° for all experiments in this study. Figure 4b presents the schematic structure of the TENG is in contact with the streamflow. The generation of triboelectric charge was explained based on the change in the electrical double layer (EDL) at the liquid-solid interface [45,46]. The EDL includes two ion-distribution regions, namely a Stern layer and a diffuse layer. In the electrostatic interface, the water is considered as a positive layer and the solid is considered as a negative layer. When a solid surface, which contains high electronegative, is in aqueous solutions, the oppositely charged ions concentrate to the surface to make the electroneutrality balanced. As a result, the immobile part is formed near the solid surface and is called the Stern layer. In contrast, the mobile part as called the diffuse layer of the EDL is formed based on the thermal motion of the aqueous solution.

According to the effect of water flow on the EDL, the working principle of the TENG is presented in Figure 4c by single-electrode mode. In this study, we used a peristaltic pump to transfer the water where an unsteady streaming flow is applied to the TENG surface and can be divided into two-state: increase and reduce the flow velocity (Figure S1, Supporting Information). In the initial state, the flow velocity starts the increasing process, the EDL is into the equilibrium state and no electric flow is generated (Figure 4c(i)). When a flow velocity increases on the surface solid, the equilibrium state of the EDL can be changed to a non-equilibrium state [47]. Although the electrostatic attraction in the Stern layer is very strong and the ions still are immobile, the ionic adsorption capacity in the Stern layer can be increased with an increase in water flow. Therefore, more positive ions are concentrated in the Stern layer, leading to the difference of positive potential between a Stern layer and a Cu electrode layer. Thus, the electrons move from the Cu electrode to the ground and generated electricity as shown in Figure 4c(ii). When the flow velocity reaches the maximum value, no more positive ions can be adsorbed in the Stern layer and the EDL layer achieve an equilibrium state (Figure 4c(iii)). After that, when the streaming flow change to reduce velocity, the EDL equilibrium is broken leading to the positive ions decrease in the Stern layer. The electron flow is organized by attracting electrons from the ground to the Cu electrode as shown in Figure 4c(iv) and finish a cycle of the triboelectrification. Then, the next cycle will be started until the flow velocity change from reduction state to increase state to generate an AC electrical output (Figure 4d).

### 3.3. Electrical Output Characterization of PSPE TENG

**Figure 5:**
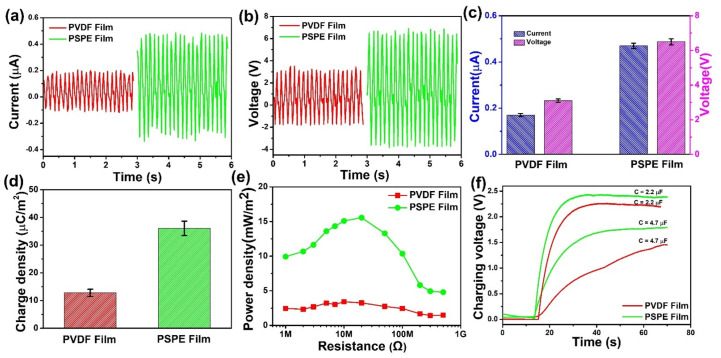
Electrical output characterization of PVDF and PSPE-TENG operated at a flow rate of 3 mL/s: (**a**) current, (**b**) voltage, (**c**) comparison of current and voltage, (**d**) charge density, (**e**) power density with loading resistances ranging from 1 MΩ to 500 MΩ, and (**f**) charging voltage curves for different capacitors.

The electrical output characterization of PVDF and PSPE-based TENG were exhibited in Figure 5 to prove the influence of the surface polarization enhancement. The TENG working conditions were set up with a water flow rate of 3.0 mL/s, a contact surface area of 20 × 20 mm^2^, and an inclination angle of 45°. The output current and voltage of the PVDF-TENG and PSPE-TENG were measured in Figure 5a,b. As the triboelectric layer of the TENG was increased surface polarization, the generated current increased from 0.17 μA to 0.47 μA, and voltage occurred from 3.1 V to 6.5 V. Furthermore, the output current of PSPE-TENG showed excellent stability and durability even after it was worked for more than 6000 cycles (Figure S2, Supporting Information). In addition, the charge density of PSPE-TENG gains the value of approximately 36.1 μC/m^2^, which is 2.8 times higher than that of PVDF-TENG (Figure 5d). As stated in the characterization of the film, the surface polarization enhancement leads to a high dielectric constant and low surface energy. The relation between transferred charge density (σ′) and dielectric constant can be presented as follows [27]:

(3) $$\sigma \prime =\frac{\sigma_{0}d_{gap}}{d_{gap}+d_{film}/\varepsilon_{film}}$$

where $d_{film}$, $\varepsilon_{film}$ are the thickness and dielectric constant of the triboelectric film, $d_{gap}$ is the gap distance and $\sigma_{0}$ is the charge density at the equilibrium state. According to this equation, the transferred charge density of the film increases with an increase of the dielectric constant. Besides, the low surface energy of the triboelectric film is the cause of the charge separation easier, which is increased transferred charge density [48]. While the output current and voltage are proportional to charge surface density; thus, the high dielectric constant and low surface energy can enhance the output performance of the TENG.

As a device for self-powered sensors, providing enough energy to the system is one of the common issues for the power supply capability of TENG. Therefore, measurement of output power density at different load resistance is the key factor to investigate that effect. As shown in Figure 5e, the maximum power density of PSPE-TENG reaches the value of 15.6 mW/m^2^ at an external load resistance of 20 MΩ, which is approximately 4.7 times higher than that of PVDF-TENG. On this basis, the TENG output is charged with various capacitors to investigate the charging speed and storage capacity (Figure 5f). For all PVDF and PSPE based TENG, the charging voltage curves are rapidly increased at the beginning, and then they gradually decrease. The PSPE-TENG can obtain a higher voltage and charging speed for both capacitors (2.2 μF and 4.7 μF) compared to PVDF-TENG. A literature review of the liquid-solid TENG regarding the output performance and sensing objective is displayed in Table 2. It can be seen that the previous self-powered based TENG showed great potential for different sensing objects. However, this proposes PSPE-TENG show the high-output performance, and excellent stability and durability, which are the most significant advantage for the self-powered sensors system.

### 3.4. Practicability in Self-Powered Sensing

**Figure 6:**
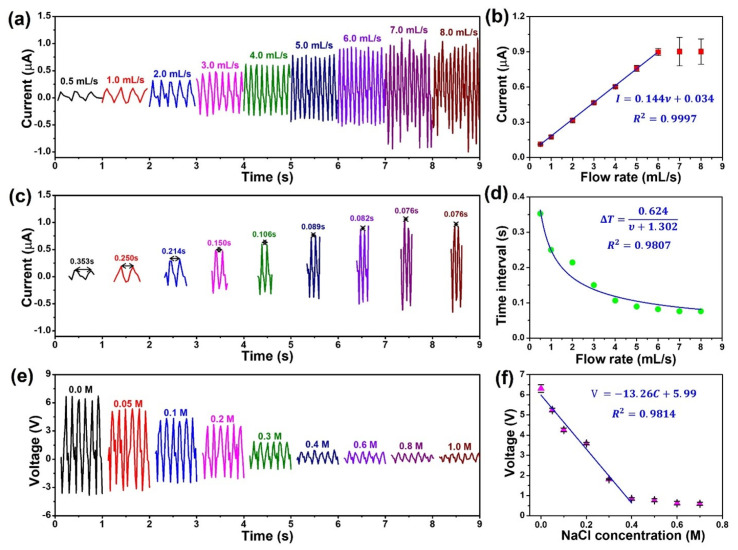
(**a**) Output current of PSPE-TENG depending on the water flow rate, (**b**) regression analysis of flow rate based on output current; (**c**) calculate the time interval between two peaks of current working on different water flow rates, (**d**) regression analysis of flow rate based on time interval; (**e**) output voltage of PSPE-TENG depending on NaCl concentration, (**f**) regression analysis of NaCl concentration based on output voltage.

According to a high-output performance and good stability, the PSPE-TENG has the potential application in streamflow sensing. Figure 6a presented the output current of PSPE-TENG depending on the water flow rate from 0.5 mL/s to 8 mL/s. It is noted that the output current gradually increases from 0.11 μA to 0.90 μA as the water flow rate enhances from 0.5 mL/s to 6 mL/s. On the contrary, upon further raising of water flow rate (up to 8 mL/s), the output current is no obvious change, and the output peaks tend to decrease stability. The effect of water flow rate on the output performance of TENG was presented in the working principal part. When the velocity of water flow increases, the number of ion adsorption on the triboelectric layer also increases, hence enhancing the electrical power output. However, the ionic adsorption capacity in the Stern layer reached the limit if the flow rate continues to increase, resulting in no increment of output performance. From this result, regression analysis of flow rate based on output current is determined in Figure 6b, which demonstrates a strong linear relationship between the current and flow rate from 0.5 mL/s to 6 mL/s. Therefore, the regression equation is given by:

(4) I=0.144υ+0.034

where I is the output current (μA), υ is the velocity of water flow (mL/s). The fitting relation is obtained with high sensitivity $k=0.144\;\mu A/\mathrm{mL}\cdot s^{-1}$ and correlation coefficient $R^{2}=0.9997$. Moreover, the increased velocity of water flow could reduce the charging and discharging time of the TENG, which is decreased the time interval between two cycles (ΔT) of output current (Figure 6c). The curve fitting of the data is shown in Figure 6d and follow the relationship:

(5) $$\Delta T=\frac{0.624}{\upsilon +1.302}$$

This regression analysis shows a high correlation coefficient $R^{2}=0.9807$, while the water flow rate increase from 0.5 mL/s to 8 mL/s, suggesting that the velocity of streamflow could be calculated without regard to the amplitude of the output performance. This implies that the PSPE-TENG can sense the velocity of different aqueous solutions with high sensitivity and accuracy. According to this phenomenon, the PSPE-TENG could be applied as a multifunction sensor system to sensing different objectives, such as flow rate, ion concentration, and ion detection at the same time. Therefore, the ability for ion concentration sensing of this TENG was investigated by changing the salinity of the aqueous solutions as shown in Figure 6e. The proportional relationship between ion concentration (C) and triboelectric charge generation ($Q_{d}$） as follows [32]:

(6) $$Q_{d}\propto -logC\equiv pC$$

According to this equation, the triboelectric charge generation is linearly proportional to pC, which is a conception of pH. The plot Figure S3 (Supporting Information) is shown the regression analysis of pC based on output voltage to confirm such phenomenon. Thus, the output performance of TENG tends to decrease with a higher ion concentration [51,52,53], which has the same trend line as output experiment data. The output voltage of PSPE-TENG gradually decease from 6.3 V to 0.6 V as the NaCl concentration change from 0 M to 0.7 M. As expected, the relationship between the output voltage and salinity could be linearly fitted, when the NaCl concentration is in the range of 0 M to 0.4 M, and follow the equation:

(7) V=−13.26C+5.99

where V is the output voltage of PSPE-TENG, and C is the NaCl concentration. In this part, high sensitivity $k=-13.26\;V/\mathrm{mol}\cdot L^{-1}$ and correlation coefficient $R^{2}=0.9814$ are obtained. Considering the output tendency with the presence of NaCl in water, the PSPE-TENG can be expanded as a self-powered sensor to determine further specific ion concentrations in an aqueous solution. Additionally, following other advantages of TENG device such as simple structure, low cost, flexibility, and miniaturization, the PSPE-TENG is a good potential candidate for application in self-powered streamflow sensors.

## 4. Conclusions

In summary, we have fabricated the high-output liquid-solid TENG based on PVDF surface polarization enhancement film as a triboelectric layer. By introducing a high negative fluorine function group on the film surface, the dielectric constant and hydrophobic properties were increased, which can be demonstrated by measuring the optical and electrical properties of the film. This implied the polarization as well as increased triboelectric charge transfer capability, which plays a critical role in significantly improving the TENG output performance. Thus, the output current and voltage of the PSPE-TENG reach a value of 0.47 μA and 6.5 V, which are 2.8 and 2.2 times higher than the corresponding values of the PVDF-TENG under the flow rate of 3 mL/s, respectively. The maximum power density increased by about 4.7 times and obtained 15.6 mW/m^2^. On the other hand, the PSPE-TENG-driven sensor system was shown a strong linear relationship, resulting in the high sensitivity ($0.144\;\mu A/\mathrm{mL}\cdot s^{-1}$ with flow rate and $-13.26\;V/\mathrm{mol}\cdot L^{-1}$ with NaCl concentration) and minimal error detection. Moreover, by calculating the time interval between two cycles of output current, the flow rate of the stream could be determined regardless of the amplitude of the output result. Based on its high sensitivity and output performance, the PSPE-TENG was demonstrated as a potential candidate to develop the self-powered sensor in streamflow research.

## Figures and Tables

**Table 1:** Elemental compositions of PVDF and PSPE film.

Sample	Atom Percent (%)			
	C1s	O1s	F1s	Si2p
PVDF	50.70	0.45	48.85	0.00
PSPE	41.39	3.64	53.39	1.58

**Table 2:** The comparison of liquid-solid TENG regarding the output performance and sensing objective.

Triboelectric Layer	Current (μA)	Voltage (V)	Charge Density (μC/m^2^)	Power Density (mw/m^2^)	Sensing Objective	References
Hydrophobic fumed silica film	0.002	~	4.0	~	Biomedical sensing	[30]
PDMS channel	0.01	0.02	2.6	0.01	Pressure fluctuation	[49]
Thin PDMS layer	0.012	0.019	~	~	Pressure, finger motion	[15]
PTFE tube	0.23	2.3	1.2	0.15	Velocity transducing	[50]
FEP U-shaped tube	0.26	81.7	28.9	~	Ion concentration	[51]
PVDF film	0.17	3.1	12.7	3.3	Velocity, ion concentration	This work
PSPE film	0.47	6.5	36.1	15.6	Velocity, ion concentration	This work

## Data Availability

The authors confirm that the data supporting the findings of this study is available within the article.

## Supplementary Materials

The following supporting information can be downloaded at: https://www.mdpi.com/article/10.3390/polym14050960/s1, Figure S1: Schematic diagram depicting the change in the EDL at a triboelectric layer in aqueous solution in different velocity; Figure S2: The stability of PSPE-TENG operated at a flow rate of 3 mL/s for about 6000 cycles; Figure S3: The regression analysis of pC based on the output voltage.
