# Supplementary material for: Polyvinylidene Fluoride Surface Polarization Enhancement for Liquid-Solid Triboelectric Nanogenerator and Its Application
Source: Polymers (Basel). 2022 Feb 28;14(5):960. doi: 10.3390/polym14050960 (PMC8912612; doi:10.3390/polym14050960)
Supplement: Supplementary file 1 [file polymers-14-00960-s001.zip › polymers-1596871-Supplementary.pdf]

polymers

MDPI

Supplementary Material

Polyvinylidene Fluoride Surface Polarization Enhancement for Liquid-Solid Triboelectric Nanogenerator and Its Application

Duy Linh Vu, Chau Duy Le and Kyoung Kwan Ahn *

Fluid Power & Machine Intelligence (FPMI) Laboratory, School of Mechanical Engineering, University of Ulsan, 93, Daehak-ro, Nam-gu, Ulsan 44610, South Korea; vuduylinhbk@gmail.com (D.L.V); lechauduy@gmail.com (C.D.L); kkahn@ulsan.ac.kr (K.K.A)

* Correspondence: kkahn@ulsan.ac.kr

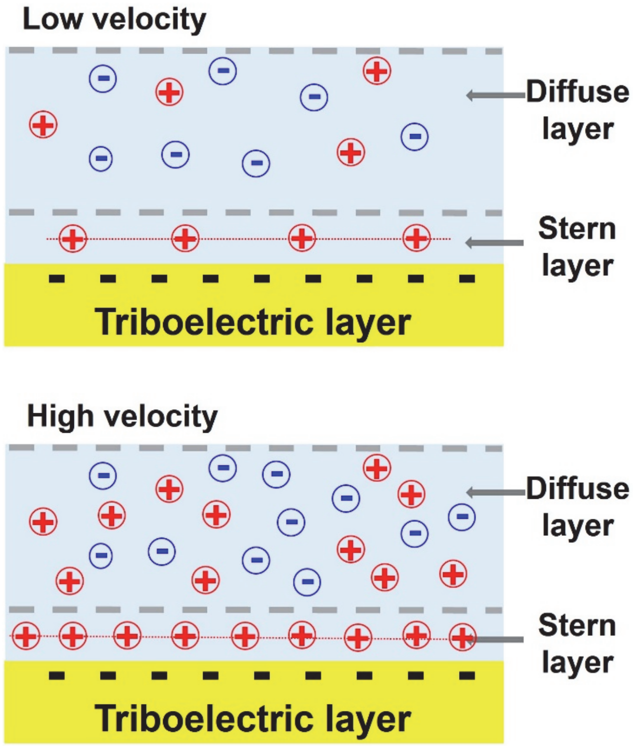

Figure S1. Schematic diagram depicting the change in the EDL at a triboelectric layer in aqueous solution in different velocity

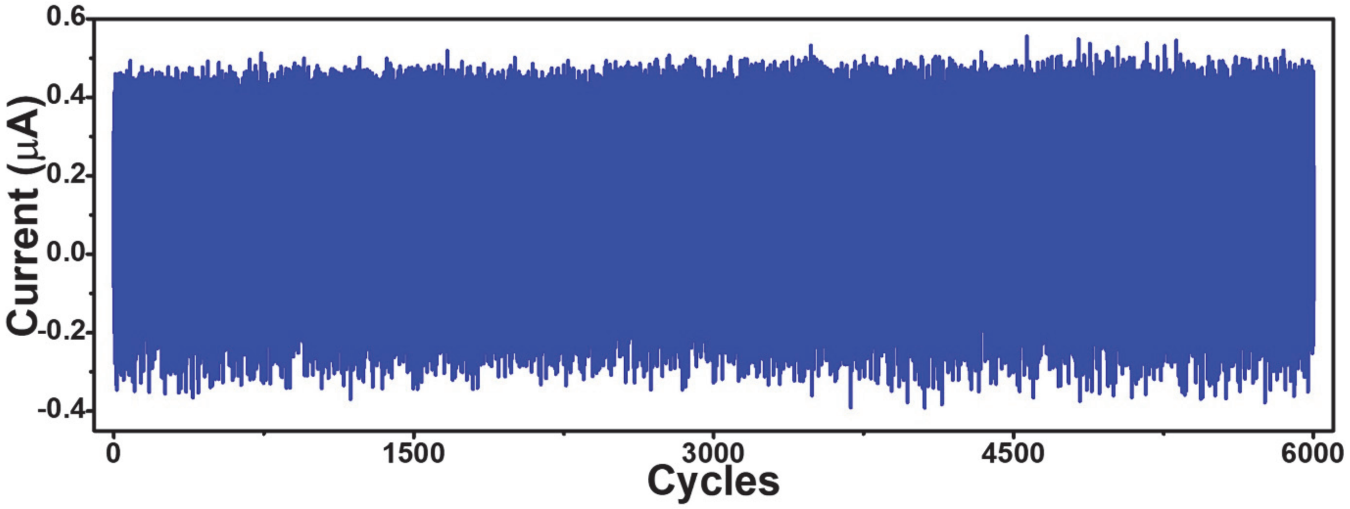

Figure S2. The stability of PSPE-TENG operated at a flow rate of 3 ml/s for about 6,000 cycles

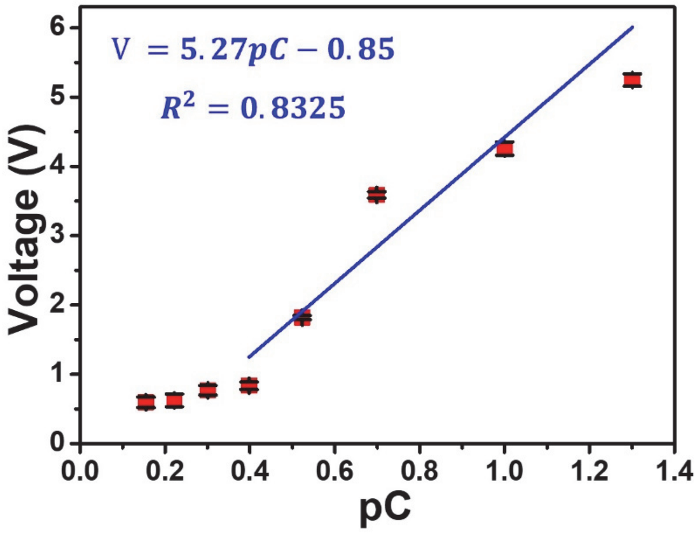

Figure S3. The regression analysis of pC based on the output voltage
